# Mechanisms and Effects of a WeChat-Based Intervention on Suicide Among People Living With HIV and Depression: Path Model Analysis of a Randomized Controlled Trial

**DOI:** 10.2196/14729

**Published:** 2019-11-27

**Authors:** Yiran Li, Yan Guo, Y Alicia Hong, Mengting Zhu, Chengbo Zeng, Jiaying Qiao, Zhimeng Xu, Hanxi Zhang, Yu Zeng, Weiping Cai, Linghua Li, Cong Liu

**Affiliations:** 1 Department of Epidemiology and Biostatistics School of Public Health Sun Yat-sen University Guangzhou China; 2 Sun Yat-sen Center for Migrant Health Policy Guangzhou China; 3 Sun Yat-sen Center for Global Health Institute of State Governance Guangzhou China; 4 Department of Health Administration and Policy College of Health and Human Services George Mason University Fairfax, VA United States; 5 South Carolina SmartState Center for Healthcare Quality Arnold School of Public Health University of South Carolina Columbia, SC United States; 6 Department of Health Promotion Education, and Behavior, Arnold School of Public Health University of South Carolina Columbia, SC United States; 7 National Center of AIDS/STD Control and Prevention Chinese Center for Disease Control and Prevention Beijing China; 8 Department of Infectious Diseases Eight People’s Hospital Guangzhou China

**Keywords:** HIV, mHealth, depression, suicide

## Abstract

**Background:**

People living with HIV and depression have high rates of suicide. Studies of mobile health (mHealth) interventions have shown feasibility, acceptability, and efficacy in improving mental health in people living with HIV and depression. However, few studies have examined the mechanisms and effects of mHealth interventions on suicide.

**Objective:**

This study was designed to examine the mechanisms and effects of a WeChat-based intervention, *Run4Love*, on suicide among people living with HIV and depression in China, while considering perceived stress and depressive symptoms as mediators.

**Methods:**

A sample of 300 People living with HIV and depression was recruited from the outpatient clinic of a large HIV or AIDS treatment hospital and was randomized to the *Run4Love* group or a control group. Data were collected at baseline, 3-, 6-, and 9-month follow-ups. Path analysis modeling, with longitudinal data, was used in data analyses.

**Results:**

The *Run4Love* mHealth intervention had a direct effect on reducing suicide rate at the 6-month follow-up (beta=−.18, *P*=.02) and indirect effect through reducing perceived stress and depressive symptoms at the 3-month follow-up (beta=−.09, *P*=.001). A partial mediating effect between perceived stress and depressive symptoms accounted for 33% (–0.09/–0.27) of the total effect.

**Conclusions:**

Through path analyses, we understood the mechanisms and effects of an mHealth intervention on suicide prevention. The findings underscored the importance of stress reduction and depression treatment in such a program. We call for more effective suicide prevention, especially mHealth interventions targeting the vulnerable population of people living with HIV and depression.

**Trial Registration:**

Chinese Clinical Trial Registry ChiCTR-IPR-17012606; http://www.chictr.org.cn/showprojen.aspx?proj=21019

## Introduction

### Background

People living with HIV have high rates of depressive symptoms. For example, a nationally representative survey in the United States reported that 36% of people living with HIV had depression [1]. In China, the rate of depression among people living with HIV was 50.8%, according to a recent review study [2]. Out of 37.9 million people living with HIV in the world [3], more than 12 million are living with depression [4]. The literature has shown a causal relationship between depression and suicide [5,6]. Previous studies have also reported high suicide rates in people living with HIV. A survey of 1560 people living with HIV in the United States revealed that 26% of the participants had a suicidal ideation and 13% had a suicidal attempt [7]. According to a recent survey in China, 32.4% of people living with HIV had had suicidal ideation or behavior since HIV diagnosis [8]. Such high rates of suicide and depression warrant effective interventions targeting the vulnerable population of people living with HIV and depression, especially the interventions that can reach a large number of people living with HIV and depression efficiently, such as mobile health (mHealth) interventions.

### Reviews

According to a recent systematic review, the majority of the existing mHealth interventions targeting people living with HIV were focused on medication adherence or retention in care [9]. Only a small number of mHealth interventions were implemented to improve mental health outcomes or decrease suicidal risks among people living with HIV and depression [9-11]. In a pilot mHealth intervention, Swendeman and colleagues developed a self-monitoring program, which showed initial efficacy in reducing stress in people living with HIV [12]. Van Luenen and colleagues tested a Web-based cognitive behavioral therapy program via a randomized controlled trial (RCT) with 188 people living with HIV and depression. The intervention successfully reduced depressive symptoms and anxiety of the intervention group at 8-week and 12-week follow-ups compared with the control group [13]. Despite the growing interest and initial efficacy of mHealth interventions to deliver mental health services and prevent suicide among people living with HIV and depression, no study existed on the potential mechanism of the intervention in mental health outcomes, especially on the basis of a longitudinal design.

### Aims

Accordingly, in this study, we aimed to examine the mechanisms of the mHealth intervention *Run4Love* in a suicide outcome in a sample of people living with HIV and depression by conducting path model analyses, using longitudinal data from an RCT [14]. The *Run4Love* was a WeChat-based intervention, comprising 2 major components: the adapted cognitive-behavioral stress management (CBSM) course and physical activity promotion. It was designed to reduce stress through multiple coping strategies.

**Figure 1:**
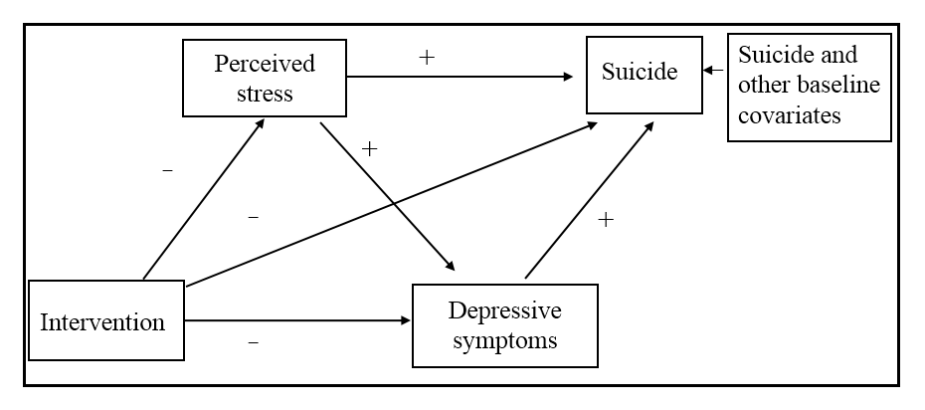
Hypothesized path model of intervention, perceived stress, depressive symptoms, and suicide in people living with HIV and depression.

The literature has documented the importance of stress reduction in depression prevention [15-18]. Previous studies also showed that depressive symptoms mediated perceived stress and suicidal behaviors [19-21]. However, no study has investigated the mechanisms of stress and depressive symptoms in suicide in the context of mHealth intervention. On the basis of the associations among perceived stress, depressive symptoms, and suicide illustrated in the previous studies [19-21], we hypothesized that perceived stress and depressive symptoms at a 3-month follow-up served as mediators on the effects of the mHealth intervention on suicide at a 6-month follow-up. The hypothesized model is depicted in Figure 1.

## Methods

### Research Setting

A total of 300 participants were recruited from an outpatient clinic of the only designated hospital for HIV or AIDS treatment in Guangzhou, China. As the capital city of Guangdong Province, Guangzhou is the third largest city in China. According to the AIDS Prevention and Treatment Report in 2017, more than 53,600 people living with HIV live in Guangdong province [22].

### Participant Eligibility and Recruitment

We recruited participants in the waiting room of an outpatient clinic. A member of the research team invited patients to participate in a research study. Those who showed initial interest were invited to a private space for further explanation and screening. The participation criteria included the following: (1) at least 18 years old, (2) HIV-seropositive status (registered in the hospital system or with an official document), (3) having clinically significant depressive symptoms (Center for Epidemiologic Studies-Depression; CES-D score≥16), and (4) having a mobile phone and WeChat account. Patients were excluded if they were (1) currently under psychiatric or psychological treatment, (2) unable to finish the questionnaire, (3) unable to read or listen to the materials on WeChat, and (4) unable to engage in physical activities because of medical reasons.

Participants who met the eligibility criteria and provided written informed consent were invited to participate. They completed a baseline survey before they were randomized to the intervention group or control group of the *Run4Love* trial. All participants received breakfast (milk and bread) as an incentive. The study protocol was approved by the Institutional Review Board of the Sun Yat-sen University.

### The *Run4Love* Intervention

**Figure 2:**
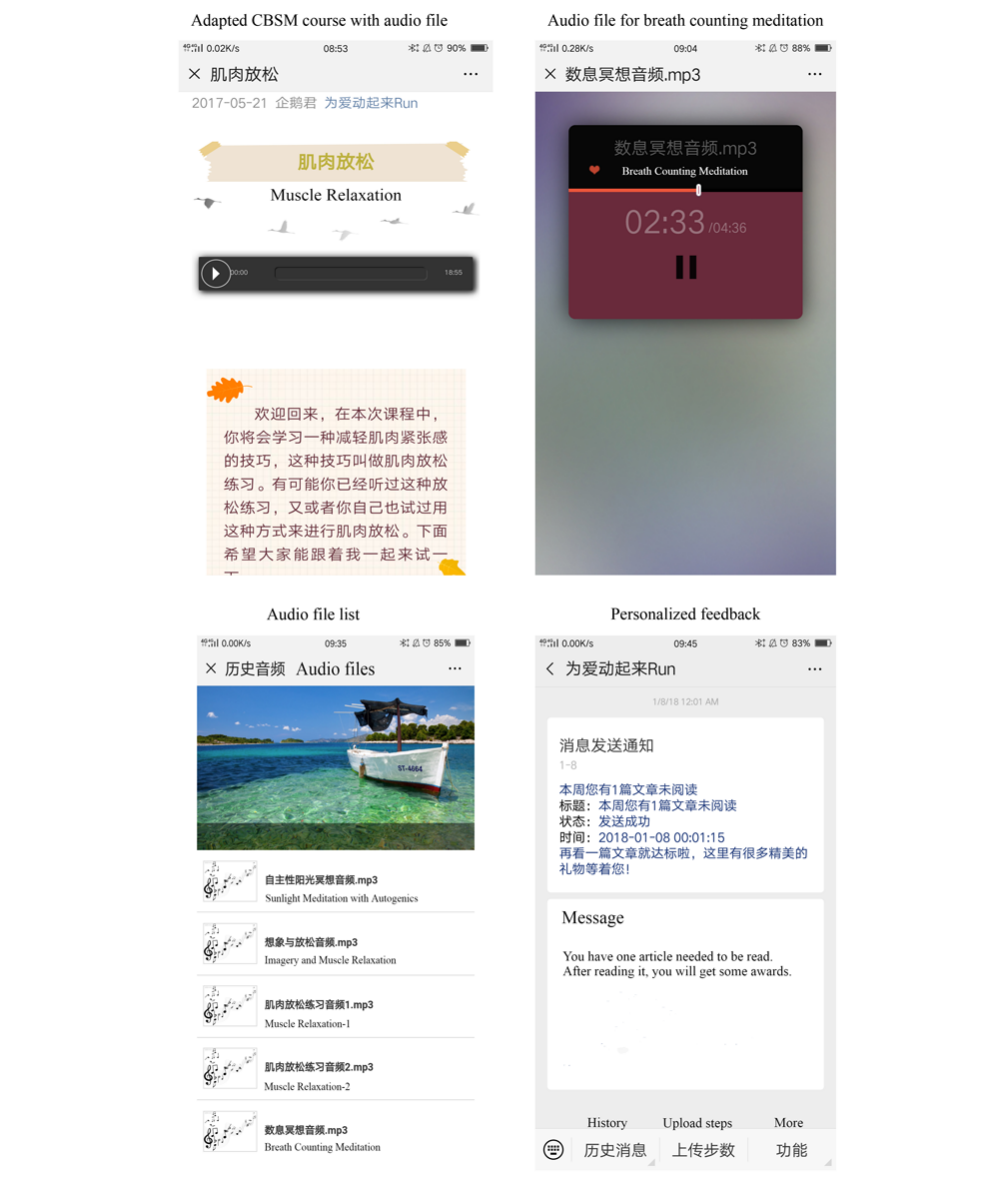
WeChat users’ interfaces in the Run4Love Program. CBSM: cognitive-behavioral stress management.

The *Run4Love* intervention was adapted from the CBSM program [23], which was designed as a face-to-face intervention program for people living with HIV, and it has demonstrated good efficacy in various populations and settings [24-26]. We adapted the CBSM into a multimedia format and delivered it via WeChat, the most popular social media platform in China, with over 1 billion active users [27]. The intervention protocol is detailed elsewhere [14]. Briefly, participants assigned to the intervention group received a series of adapted CBSM courses, and they were encouraged to do regular physical activities for 3 months. Multimedia information, with automatic progress tracking and personalized feedback, was delivered via the *Run4Love* WeChat account (Figure 2). The main purpose of the program was to alleviate people living with HIV’s depressive symptoms and improve their quality of life by reducing perceived stress. Participants in the wait-list control group received a brochure on HIV-related nutrition. Outcomes were assessed at baseline, 3, 6, and 9 months. All participants in the control group would receive the intervention after the completion of the trial.

### Measurements

#### Demographic Characteristics

Participants’ demographic characteristics assessed in the study included age, gender, sexual orientation, marital status, education, and duration since HIV diagnosis.

#### Depressive Symptoms

Depressive symptoms were assessed using the Chinese version of the CES-D scale at baseline and each follow-up [28]. The scale has been validated in various Chinese chronic patients, and it demonstrated high levels of validity and reliability [29,30]. The CES-D comprises 20 items, such as *My appetite was poor* and *I could not shake off the blues.* Participants were asked to select the frequencies of physiological symptoms during the past week. The total scores ranged from 0 to 60, with a higher score indicating a higher level of depressive symptoms. Participants with scores of 16 or higher were considered as having clinically significant depressive symptoms [31]. The Cronbach alpha of the scale was .77 in this study.

#### Perceived Stress

Perceived stress was assessed with the 10-item Chinese version of Perceived Stress Scale (PSS-10) at baseline and each follow-up [32]. The scale was the most widely used instrument to measure perception of stress; the validity and reliability of the scale had been validated and established in the Chinese population [33-35]. Participants were asked about their feelings and thoughts in the past month (eg, *How often have you felt nervous and stressed*?). The total scores of the scale ranged from 0 to 40, with higher scores indicating higher levels of perceived stress. Scores ranging from 0 to 13, 14 to 26, and 27 to 40 were considered as low, moderate, and high levels of perceived stress, respectively [36]. In this study, the Cronbach alpha of the PSS-10 was .67.

#### Suicide

Suicide was defined as having had suicidal ideation or attempt in this study. Suicidal ideation referred to the planning or thinking of committing suicide, and suicidal attempt was the actual behavior of committing suicide. Participants were asked how many times they had had thought about committing suicide and how many times they had actually attempted suicide in the past 3 months at baseline and each follow-up. Participants who answered *yes* to either of the 2 questions were considered as having had suicide. Those who answered *no* to both of the questions were considered as not having had suicide (dummy coded as 0=not having had suicide, 1=having had suicide).

### Data Analysis

First, descriptive analyses were used on demographic characteristics, perceived stress, depressive symptoms, and suicide. The continuous variables with skewed distribution (eg, age, duration since HIV infection, perceived stress, and depressive symptoms) were described using median (interquartile range; IQR), and categorical variables were described using frequencies and percentages.

Second, treatment group comparisons for the outcome and mediating variables at 3 assessment points were conducted using Wilcoxon rank-sum test for continuous variables with skewed distribution (eg, perceived stress and depressive symptoms) and Chi-square test for categorical variables (eg, suicide). Third, bivariate analyses of suicide were performed using Wilcoxon rank-sum test (for continuous variables with skewed distribution) and Chi-square test (for categorical variables).

To further examine the mechanisms of how participants’ suicide changed as a result of the intervention, we conducted a path analysis after controlling for the baseline suicide and potential confounders, such as demographics. A mediation model of the path analysis was used to test the hypothesis whether the intervention effect on suicide could be explained by the mediating factors of perceived stress and depressive symptoms. Such modeling was designed to explore the mechanisms and direct and indirect effects of the intervention on suicide. The pathways of intervention→suicide, intervention→depressive symptoms→suicide, intervention→perceived stress→suicide, and intervention→perceived stress→depressive symptoms→suicide were examined, respectively (dummy coded as 0=control group, 1=intervention group). The statistical significance was defined as *P*<.05.

The model was estimated by weighted least squares means and variance–adjusted estimation. Multiple indicators were used to evaluate the goodness of fit of the model, including Chi-square statistic, Comparative Fit Index (CFI), Tucker-Lewis Index (TLI), root mean square error of approximation (RMSEA), and weighted root mean square residual (WRMR). Smaller Chi-square value indicates better model fit. CFI≥0.95, TLI>0.90, RMSEA≤0.06, and WRMR≤1.00 indicate good model fit [37,38]. Descriptive statistics, bivariate statistics, and correlation analyses were performed using SAS version 9.4 (SAS Institute, Inc). Path analysis was performed using Mplus version 7.0 (Muthen and Muthen) [39].

## Results

### Baseline Characteristics of the Participants

As shown in Table 1, the median (IQR) age of the 300 participants was 27.5 (24.5, 31.3) years, with a range from 18 to 51. Most (277/300, 92.3%) of the participants were male. More than four-fifths (245/300, 81.7%) of the participants were homosexual, bisexual, or uncertain of their sexual orientations, and 87.3% (262/300) were unmarried. About 60.7% (182/300) of the participants had completed at least some college education. The median duration of HIV infection was 1.7 years (IQR 0.6, 3.8).

At baseline, a majority (256/300, 85.4%) of the participants had a moderate level of perceived stress, followed by 8.3% (25/300) having a high level and 6.3% (19/300) a low level of stress. The median (IQR) of the CES-D score was 23.0 (19.0, 28.0). Approximately 44.0% (132/300) of the participants reported having considered committing suicide and 9.7% (29/300) having tried to attempt suicide in the past 3 months. In total, about 45.0% (135/300) of the participants had suicidal ideation or attempts in the past 3 months.

The proportion of homosexual, bisexual, or sexual orientation–uncertain participants in the intervention group was slightly higher than the control group (130/150, 86.7% vs 115/150, 76.7%; *P*=.03). Other demographic characteristics, mental health outcomes (depressive symptoms and perceived stress), and suicide were balanced in the 2 groups at baseline.

**Table 1:** Sample characteristics of people living with HIV and depression (N=300).

Characteristics		Value
Age (years), median (IQR^a^)		27.5 (24.5, 31.3)
**Gender, n (%)**		
	Male	277 (92.3)
	Female	23 (7.7)
**Sexual orientation, n (%)**		
	Heterosexual	55 (18.3)
	Homosexual/bisexual/uncertain	245 (81.7)
**Education, n (%)**		
	≤High school	118 (39.3)
	>High school	182 (60.7)
**Marital status, n (%)**		
	Single	262 (87.3)
	Married	38 (12.7)
**Employment status, n (%)**		
	Unemployed	49 (16.3)
	Employed	251 (83.7)
Duration since HIV diagnosis (years), median (IQR)		1.7 (0.6, 3.8)
Perceived stress, median (IQR)		20.0 (18.0, 23.0)
**Levels of perceived stress, n (%)**		
	Low perceived stress	19 (6.3)
	Moderate perceived stress	256 (85.3)
	High perceived stress	25 (8.3)
Depressive symptoms, median (IQR)		23.0 (19.0, 28.0)
**Suicidal ideation (times in the last 3 months), n (%)**		
	0	168 (56.0)
	1-2	84 (28.0)
	≥3	48 (16.0)
**Suicidal attempt (times in the last 3 months), n (%)**		
	0	271 (90.3)
	1-2	19 (6.3)
	≥3	10 (3.3)
**Suicide (in the last 3 months), n (%)**		
	Yes	135 (45.0)
	No	165 (55.0)

^a^IQR: interquartile range.

### Changes in Outcomes and Mediating Variables Over Time

The intervention had significant effects on the mental health outcomes over time. A total of 274 participants (91.3%, 274/300) remained at the 3-month follow-up (139 in the intervention group; 135 in the control group), and 265 (88.3%, 265/300) participants remained at the 6-month follow-up (132 in the intervention group; 133 in the control group). As reported in Table 2, the median score of the perceived stress was 20 at baseline in the intervention and control groups; it changed to 17 in the intervention group compared with 19 in the control group at the 3-month follow-up. Similarly, the depressive symptoms in the intervention group reduced from 23 at baseline to 17 at the 3-month follow-up, whereas in the control group, it remained unchanged at 23. The rate of clinically significant depressive symptoms reduced from 100% to 56.8% (79/139) in the intervention group compared with 79.3% (107/135) in the control group at the 3-month follow-up. The rate of suicide reduced from 44.0% (66/150) at baseline to 31.7% (44/139) at the 3-month follow-up, and it further reduced to 21.2% (28/132) at the 6-month follow-up in the intervention group. The difference in the rate of suicide between the 2 groups was close to 20% (21.2% in intervention group vs 40.6% in control group) at 6 months. The intervention group had improved significantly in 3 variables—perceived stress at the 3- and 6-month follow-ups (both *P*<.001), depressive symptoms at the 3- and 6-month follow-ups (both *P*<.001), and suicide at the 6-month follow-up (*P*<.001).

**Table 2:** Mental health and behavior outcomes at baseline and follow-ups.

Variables	Baseline (T_0_)			3 months (T_1_)			6 months (T_2_)		
	I^a^ (n=150)	C^b^ (n=150)	*P* value	I (n=139)	C (n=135)	*P* value	I (n=132)	C (n=133)	*P* value
Perceived stress, median (IQR^c^)	20 (17, 22)	20 (18, 23)	.14^d^	17 (12, 20)	19 (16, 22)	<.001^d^	17 (13, 20)	19 (16, 23)	<.001^d^
Depressive symptoms, median (IQR)	23 (19, 28)	23 (19, 27)	.81^d^	17 (11, 24)	23 (18, 31)	<.001^d^	17 (10, 23)	24 (16, 32)	<.001^d^
Clinically significant depressive symptoms, n (%)	150 (100.0)	150 (100.0)	—^e^	79 (56.8)	107 (79.3)	<.001^f^	68 (51.5)	102 (76.7)	<.001^f^
Suicide, n (%)	66 (44.0)	69 (46.0)	.82^f^	44 (31.7)	49 (36.3)	.42^f^	28 (21.2)	54 (40.6)	<.001^f^

^a^Intervention group.

^b^Control group.

^c^IQR: interquartile range.

^d^Wilcoxon rank-sum test.

^e^Chi-square test is not applicable in this cell. All participants had depressive symptoms in both the intervention and control groups.

^f^Chi-square test.

### Bivariate Analysis of Suicide

Results of bivariate analyses between demographic characteristics and suicide indicated that only employment was significantly associated with suicide. Specifically, employed participants reported lower rates of suicide than the unemployed (104/251, 41.4% vs 31/49, 63.3%, *P*=.005). Therefore, employment should be controlled as a covariate in the hypothesized path model.

### Path Model

After 3 months of the *Run4Love* intervention, 16 (5.3%) participants were lost to follow-ups, without their data of perceived stress and depressive symptoms at the 3- and 6-month assessments. The characteristics of the 16 participants were not significantly different from the remaining participants. The path model was estimated using data from the remaining 284 participants. The hypothesized model was examined, and there were 2 significant pathways: intervention→suicide and intervention→perceived stress→depressive symptoms→suicide. In this model, perceived stress did not appear to be significantly related to suicide (*beta*=−.05, *P*=.71), and the intervention did not affect depressive symptoms directly either (*beta*=−.06, *P*=.08). In addition, both employment and suicide at baseline were controlled as potential confounders in the hypothesized model, whereas only suicide showed statistical significance (*beta*=.44, *P*<.001). Consequently, employment as a covariate and the 2 nonsignificant pathways were removed in the final model.

**Figure 3:**
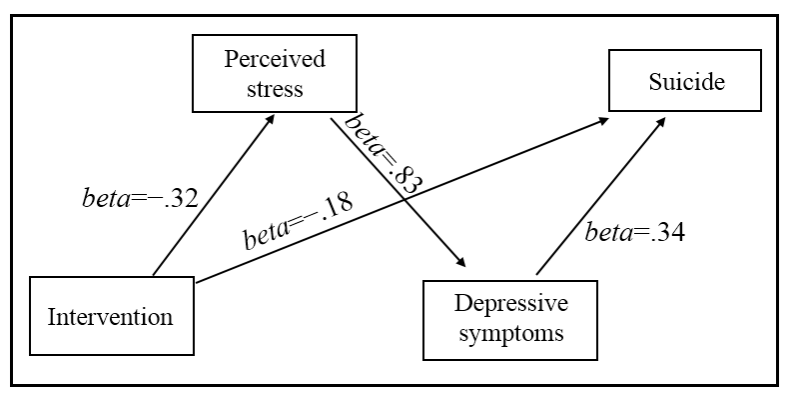
Estimation of the final path model of intervention, perceived stress, depressive symptoms, and suicide in people living with HIV and depression.

The final path model showed good model fit (*χ^2^*_4_= 6.1, *P*=.19, CFI=0.99, TLI=0.97, RMSEA=0.04, and WRMR=0.55). Standardized regression coefficients for the final model are reported in Table 3 and Figure 3. Results indicated that the intervention reduced participants’ perceived stress at the 3-month follow-up (*beta*=−.32, *P*<.001), which was positively associated with depressive symptoms at the 3-month follow-up (*beta*=.83, *P*<.001); reduced depressive symptoms consequently resulted in reduced suicide at 6 months (*beta*=.34, *P*<.001). The pathway from perceived stress to depressive symptoms had the strongest effect size in the mediation model (*beta*=.83, *P*<.001).

The direct, indirect, and total effects of the path model are summarized in Table 3. The direct effect of the intervention on suicide at the 6-month follow-up was significant (*beta*=−.18, *P*=.02). The indirect effect of the intervention on suicide via perceived stress and depressive symptoms was also significant (*beta*=−.32*.83*.34=−.09; *P*<.001). In summary, there was a partial mediating effect of perceived stress and depressive symptoms, accounting for 33% (−0.09/−0.27) of the total effect of the intervention on suicide.

**Table 3:** Coefficients of the pathways in the final model (n=284).

Pathways		Coefficient (beta)	Standardized coefficient (beta)	95% CI	SE	*P* value
Intervention→ perceived stress^a^		−3.67	−.32	−5.03 to −2.31	0.69	<.001
Intervention→ suicide^b^		−.41	−.18	−0.76 to −0.07	0.18	.02
Perceived stress^a^ → depressive symptoms^a^		1.44	.83	1.31 to 1.57	0.07	<.001
Depressive symptoms^a^ → suicide^b^		.04	.34	0.02 to 0.06	0.01	<.001
Total effect		−.62	−.27	−0.96 to −0.28	0.17	<.001
**Direct effect**						
	Intervention→ suicide^b^	−.41	−.18	−0.76 to −0.07	0.18	.02
**Indirect effect**						
	Intervention→ perceived stress^a^→ depressive symptoms^a^ → suicide^b^	−.21	−.09	−0.33 to −0.09	0.06	.001

^a^3-month follow-up.

^b^6-month follow-up.

## Discussion

### Principal Findings

This study was among the first efforts to explore the mechanisms of how an mHealth intervention reduced suicide in people living with HIV and depression, with a longitudinal design. Understanding the mechanisms is important to discern the processes between an intervention and mental health outcomes [40,41]. Better understanding of such mechanisms can facilitate targeted mHealth intervention to prevent suicide among people living with HIV and depression. Our data analyses revealed that the mHealth intervention had both direct and indirect effects on suicide, suggesting that the mHealth intervention had effectively reduced the participants’ suicide. These findings demonstrated that suicide in people living with HIV and depression could be reduced through a WeChat-based CBSM intervention, such as *Run4Love*. The mechanisms of the indirect effects were mediated through reduced perceived stress and depressive symptoms, which consequently resulted in reduced suicide.

### Implications of the Path Model

Consistent with the existing literature [15-18], perceived stress had a strong positive association with depressive symptoms, as evidenced by the large effect size shown in this study. This finding underscored the importance of reducing perceived stress to alleviate depressive symptoms in people living with HIV and depression. Many effective mHealth interventions that targeted depressive symptoms in people living with HIV have included stress management as a main component of the intervention, such as Web-based cognitive behavioral therapy [42].

The results also indicated the causal relationship between depressive symptoms at the 3-month follow-up and suicide at the 6-month follow-up. Consistent with previous studies conducted in the general population or psychiatric outpatients [5,6], the findings demonstrated that suicide might be effectively reduced through mitigating depressive symptoms. Furthermore, this study confirmed this relationship in the vulnerable population of people living with HIV and depression, who needed targeted intervention because of high rates of suicide. Some studies screened people living with HIV for depression before targeted intervention, and these demonstrated good efficacy [43-45]. Our data also suggested the importance of targeted suicide prevention intervention in people living with HIV and depression.

The direct effect of the mHealth intervention on suicide might also be explained by factors other than perceived stress and depressive symptoms, such as reduction of stigma and improvement of self-efficacy, positive coping, and social support. Further research is needed to better understand the roles and mechanisms of these potential factors on suicide.

Another finding of this study that needs to be noted is that people living with HIV and depression who committed suicide reported lower level of employment. Previous studies found that unemployment was a risk factor for suicide [46]. As a stressful event, unemployment could lead to financial distress and social isolation, which was associated with elevated stress and depression and, eventually, suicide [47,48]. Although the relationship was significant in bivariate analysis, it was no longer significant when other factors (perceived stress, depressive symptoms, and suicide at baseline) were controlled.

The data showed that the *Run4Love* mHealth intervention alone was effective, compared with the blank control group. Currently, the professional psychologists cannot meet the treatment demands of large number of people living with HIV and depression, especially in resources-limited settings, such as China. Therefore, an mHealth intervention can be an alternative resolution for people living with HIV and depression who cannot access psychotherapy. Alternatively, such an mHealth intervention can also be integrated with other traditional psychotherapies because of its easy access and adaptability. Future studies are needed to examine the effect of a stand-alone mHealth intervention compared with the one adjunct to psychotherapy.

This study suggested that public policies and coordinated efforts should be made to improve mental health outcomes, especially to reduce suicide among people living with HIV and depression. At the health care level, depression treatment and suicide prevention for people living with HIV are urgently needed. Although the guideline of HIV treatment has recommended integrating mental health screening into regular HIV care [49], such a practice was rarely implemented because of limited psychological recourses, perceived stigma of people living with HIV, and extra commute burden of face-to-face psychotherapy [42]. In middle- and low-income countries, such as China, mHealth interventions such as the WeChat-based *Run4Love* can reach more people in a cost-effective manner [13]. *Run4Love* platform also provides useful functions, such as automatic progress tracking and personalized feedback, which are necessary for an effective intervention. Our data underscore the importance of integrating mental health services into routine HIV health care by mobile apps, such as WeChat. At the community level, we call for more mHealth interventions, such as *Run4Love*, to reduce stress and improve mental health outcomes of people living with HIV and depression.

### Limitations

Several limitations of this study should be noted. First, the participants were recruited from 1 hospital in a metropolitan area, and the majority of them were men. Therefore, the results might not be generalizable to all people living with HIV and depression in China, especially women or those in rural areas. Second, the measures in this study were self-reported, with potential recall or self-report biases. We did not include a standardized measurement of suicide, and we only had 2 items about suicidal ideation and suicidal attempt. Nevertheless, self-reported suicide assessment has shown a high level of agreement with a clinician-delivered face-to-face assessment, and this could serve as an efficient and reliable method to assess suicide outcome [50]. Third, like all modeling studies, assumptions were embedded in the current path model, for example, linear relationship among variables. Finally, the pathway model included only a limited number of covariates, and other potential factors that might affect mental health outcomes were not included.

### Conclusions

In conclusion, this was the first study to examine the mechanisms of an mHealth intervention, *Run4Love*, in reducing suicide through both direct and indirect pathways, using longitudinal data from an RCT. Besides direct effect, the indirect effect was mediated by reduced perceived stress and depressive symptoms, which consequently resulted in reduced suicide. To reduce suicide in people living with HIV and depression, perceived stress and depressive symptoms are both key intervention targets. We call for targeted intervention to prevent suicide in people living with HIV and depression, especially mHealth programs that can reduce perceived stress.

## Appendix

Multimedia Appendix 1 CONSORT-EHEALTH checklist (V 1.6.1).

## Abbreviations

CBSM: cognitive-behavioral stress management
CES-D: Center for Epidemiologic Studies-Depression
CFI: Comparative Fit Index
IQR: interquartile range
mHealth: mobile health
PSS: Perceived Stress Scale
RCT: randomized controlled trial
RMSEA: root mean square error of approximation
TLI: Tucker-Lewis Index
WRMR: weighted root mean square residual
